# Immune Effector Cell-Associated HLH-like Syndrome: A Review of the Literature of an Increasingly Recognized Entity

**DOI:** 10.3390/cancers15215149

**Published:** 2023-10-26

**Authors:** Tyler Fugere, Alan Baltz, Akash Mukherjee, Mamatha Gaddam, Ankur Varma, Muthu Veeraputhiran, Cesar Giancarlo Gentille Sanchez

**Affiliations:** Winthrop P. Rockefeller Cancer Institute, University of Arkansas for Medical Sciences, Little Rock, AR 72205, USA; abaltz@uams.edu (A.B.); amukherjee@uams.edu (A.M.); mgaddam@uams.edu (M.G.); avarma@uams.edu (A.V.); veeraputhiran@uams.edu (M.V.); cgentille@uams.edu (C.G.G.S.)

**Keywords:** CAR-T cells, hemophagocytic lymphohistiocytosis, diagnostic criteria, hematologic malignancy

## Abstract

**Simple Summary:**

Chimeric antigen receptor (CAR)-T cells are a novel type of therapy that is becoming more prominent in the treatment of many hematological malignancies. They are associated with serious side effects including hemophagocytic lymphohistiocytosis (HLH), which can be fatal. Diagnosis of HLH can be challenging due to the lack of a uniform diagnostic criteria and the overlap with other frequent toxicities of CAR-T cells. Available treatments have been previously used for HLH but may not be as effective in this setting. The purpose of this literature review was to examine the evolution of the diagnostic criteria and treatment recommendations for HLH specifically in the setting of CAR-T cell therapy in order to facilitate prompt diagnosis and the implementation of suitable treatment to improve patient outcomes.

**Abstract:**

Since CAR-T cell therapy was initially approved in 2017, its use has become more prevalent and so have its side effects. CAR-T-related HLH, also named immune effector cell-associated HLH-like syndrome (IEC-HS), is a rare but fatal toxicity if not recognized promptly. We conducted a review of the literature in order to understand the prevalence of IEC-HS as well as clarify the evolution of the diagnostic criteria and treatment recommendations. IEC-HS occurrence varies between CAR-T cell products and the type of malignancy treated. Diagnosis can be challenging as there are no standardized diagnostic criteria, and its clinical features can overlap with cytokine release syndrome and active hematological disease. Suggested treatment strategies have been extrapolated from prior experience in HLH and include anakinra, corticosteroids and ruxolitinib. IEC-HS is a potentially fatal toxicity associated with CAR-T cell therapy. Early recognition with reliable diagnostic criteria and prompt implementation of treatment specific to IEC-HS is imperative for improving patient outcomes.

## 1. Introduction

Chimeric antigen receptor (CAR)-T cell therapy entails the use of T cells collected from a patient and modified ex vivo by encoding a gene with a chimeric antigen receptor against the desired antigen present on cancer cells. The T cells are expanded and then infused back into the patient as a form of autologous immunotherapy. Chimeric T cell receptors combine antibody-derived variable regions with T-cell-receptor-derived constant regions. The idea of chimeric T cell receptors was first described in 1987 by Dr. Kurosawa and his team who demonstrated that the chimeric receptor could activate T cells in response to antigens [1]. First-generation CAR-T cells were developed in 1993, and were studied in patients with metastatic ovarian cancer [2] and metastatic renal cell carcinomas [3]. However, these patients did not have any response to therapy, which was thought to be because of rapidly declining levels of the CAR-T cells in vivo. In 1998, Dr. Sadelain and his team added a CD28 receptor to provide co-stimulation to the T lymphocytes and allowed them to persist and remain active in vivo [4]. The first effective CAR-T cells (second-generation CAR-Ts) were developed in 2002 and contained both T cell receptor and CD28 signaling elements. In 2003, Dr. Sadelain et al. showed that CAR-T cells directed at CD19 and co-stimulated by CD80 and interleukin-15 were able to eradicate B-cell tumors in mice, indicating that CD19 was a promising target [5]. From 2010 to 2013, several papers were published with preliminary data regarding the safety and feasibility of CD19-directed CAR-T therapy in patients with advanced follicular lymphoma, refractory chronic lymphocytic leukemia (CLL) and relapsed B-cell acute lymphoblastic leukemia (ALL) [6,7,8,9,10,11].

The first CAR-T cell therapy approved by the FDA was tisagenlecleucel (Kymriah), which was approved on 30 August 2017, for the treatment of pediatric and young adult patients up to 25 years of age with B-ALL that is refractory or in second or later relapse. Since then, six CAR-T cell products have been approved. Four are directed against CD19 and used to treat B-cell lymphomas and ALL (Table 1), and two are directed against B-cell maturation antigens (BCMA) and used to treat multiple myeloma (Table 2). Clinical trials evaluating new targets for different hematological malignancies as well as solid tumors are ongoing. In addition, CAR-T therapy is being studied in non-malignant conditions such as systemic lupus erythematosus [12].

The most common side effects for all CAR-T products are cytokine release syndrome (CRS) and immune-effector-cell-associated neurotoxicity syndrome (ICANS). Another reported side effect of CAR-T cell therapy that is less common but serious and potentially fatal is hemophagocytic lymphohistiocytosis (HLH)/macrophage activation syndrome (MAS). When HLH occurs secondary to CAR-T cell therapy, it is commonly termed CAR-HLH. Alternatively, the American Society for Transplantation and Cellular Therapy (ASTCT) expert panel has recently coined the term “immune effector cell-associated HLH-like syndrome” (IEC-HS) to describe this phenomenon with the objective of unifying prior definitions. Recognition of IEC-HS as a biochemically and genetically distinct disorder from other previously described HLH syndromes facilitates further exploration of its distinct etiology and mechanistic features [29]. Management of this entity is of vital importance to avoid poor outcomes, but diagnosis can be difficult due to overlapping clinical presentation with other CAR-T toxicities like CRS and progressive leukemia/lymphoma.

## 2. HLH Definition and Diagnostic Criteria

Secondary HLH is an aggressive overactivation of the immune system triggered by an underlying cause. Diagnosis is made based on genetic findings, more often in children as most adults do not have an identifiable genetic mutation and usually have secondary HLH. Mortality is high, with some cohorts reporting a fatality rate as high as 57% [30,31,32]. Prior to CAR-T therapy, the common scoring systems used for diagnosing secondary HLH were the HLH-2004 criteria or the H-score. According to the HLH-2004 criteria, HLH is diagnosed if a patient has five of the following: fever, splenomegaly, cytopenias in at least two cell lineages, hypertriglyceridemia or hypofibrinogenemia with an elevated D-dimer, hemophagocytosis in bone marrow, hyperferritinemia, high levels of soluble CD25 and low or absent NK cell activity. The H-score is similar, but assigns varying point values to different criteria, including points for immunosuppression and AST elevation, and obviating soluble CD25 or NK cell activity. As Kim et al. pointed out in their review, these scoring systems have low utility in identifying possible HLH in patients after CAR-T with the HLH-2004 criteria having frequent false negatives and the H-score having a high rate of false positives. The unreliability of these scores is likely related to the fact that most patients treated with CAR-T could meet the threshold for different criteria for HLH, including elevated ferritin, fever (secondary to CRS) and cytopenias, when this is actually expected in most patients in the post-CAR-T setting who will not necessarily develop IEC-HS [33].

In 2017, Neelapu et al. formed a CARTOX Working Group and developed the criteria for diagnosing CAR-T-cell-related HLH/MAS. They noted that many of the traditional diagnostic criteria for HLH/MAS are not specific and can be present in patients with even low-grade CRS and in patients with advanced-stage hematologic malignancies. They proposed a diagnosis of HLH should be made if the ferritin levels peak at >10,000 ng/mL during the CRS phase, and if the patient develops any two of the following: grade 3 or greater organ toxicities involving the liver, kidneys, or lungs, or hemophagocytosis in the bone marrow or other organs [16]. Other groups have adopted similar criteria as well. Shah et al. used similar criteria, including peak ferritin >100,000 μg/L with at least two of the following: hepatic aminotransferases or bilirubin grade ≥ 3, creatinine grade ≥ 3, pulmonary edema grade ≥ 3 or evidence of hemophagocytosis in bone marrow aspiration/biopsy [34].

Earlier this year, the ASTCT proposed the term IEC-HS for HLH that occurs secondary to CAR-T cell therapy. It defines IEC-HS as the development of hyperinflammatory syndrome independent from CRS and ICANS that manifests with features of MAS/HLH, is attributable to immune effector cell therapy and is associated with cytopenias, hyperferritinemia, coagulopathy with hypofibrinogenemia and/or transaminitis [17]. The diagnostic criteria are listed in Table 1.

Though not specifically listing diagnostic criteria, Canna and Cron proposed that the distinction between CRS and IEC-HS is largely a difference in timing, with CRS rising and resolving within 2 weeks of CAR-T administration, while HLH-like syndrome arises after CRS and persists for possibly weeks [35]. The median onset time for HLH is reported to be 10–14 days post-infusion, usually after CRS resolution [36]. In rare circumstances, these toxicities can manifest long after the administration of CAR-T products and the initial period of close monitoring for CRS-related complications [36,37]. Awareness of the variable time in the presentation of IEC-HS and its clinical signs, which may be independent of other manifestations of CRS, in addition to optimized classification and grading systems, will help institutions and providers to more promptly and comprehensively assess for this fatal toxicity and enact appropriate treatment strategies.

## 3. HLH Incidence after CAR-T Cell Therapy

The prescribing information for both axicabtagene ciloleucel (Yescarta) and lisocabtagene maraleucel (Breyanzi) reported that HLH occurred in 0–1% of patients treated with these CAR-T cell products depending on the study [21,22,23,26,27,28]. One patient in the ZUMA-1 study had HLH with a fatal outcome [38]. The other CD19 CAR-T products outlined a higher incidence. The prescribing information for brexucabtagene autoleucel (Tecartus) reported HLH in 4% of patients with B-ALL, with a median time to onset of 8 days and a median duration of 5 days [25]. Similarly, the prescribing information for Kymriah noted that HLH occurred in 6% of patients with B-ALL with the time to onset ranging from 3 to 18 days, and in 2% of patients with diffuse large B-cell lymphoma (DLBCL) on day 7 and day 10 [18,19]. One patient with follicular lymphoma developed HLH > 1 year after receiving Kymriah with a fatal outcome [20]. None of the products report the criteria used for the diagnosis of HLH/MAS, nor do they recommend any specific treatment other than the established ones per institutional standards.

Interestingly, the rate of occurrence of IEC-HS appears to vary between products, which is perhaps related to the disease itself rather than the agent. For instance, both Kymriah and Tecartus had adverse events of IEC-HS ranging from 0 to 2% when used to treat B-cell lymphomas, but the numbers increased to 4–6% when used to treat B-ALL (Table 2). This difference in IEC-HS is not evaluable for Yescarta and Breyanzi given they were only studied in B-cell lymphomas (Table 2).

It is important to note that the real-world incidence of side effects can often differ from clinical trials. A brief review published in 2022 gave an update of the real-world incidence of IEC-HS. The authors reviewed the FDA Adverse Events Reporting System (FAERS) up to March 2022 and found that worldwide, a total of 6034 cases of IEC-HS were reported in the FAERS, resulting in an overall incidence of approximately 2% [39]. However, other reports have noted much higher rates. An abstract from the 2020 ASCO annual meeting reported 6 out of 105 patients treated for DLBCL with Yescarta developed IEC-HS. There was no significant difference in the baseline characteristics, disease stage, international prognostic index or inflammatory markers at baseline between the groups, with the exception of platelet count, which was lower in the HLH-like syndrome group [40]. Hines et al. describe a cohort of 27 pediatric and young adult patients treated with CD-19 CAR-T cell therapy in which 14.8% (n = 4) patients developed IEC-HS when applying the HLH diagnostic criteria outlined by Shah [41].

## 4. Emerging Non-CD19 CAR-T Products and Targets

Immune effector cell-related toxicity information for non-CD19-targeted CAR-T products is now emerging with insight into the frequencies of adverse events, including IEC-HS. At this time, there are two FDA-approved CAR-T cell products for the treatment of relapsed refractory multiple myeloma that target BCMA, a preserved surface antigen of plasma cells and multiple myeloma cells. Idecabtagene vicleucel (Abecma) received FDA approval based on the result of the phase 2 KarMMA study with long-term efficacy and safety data reporting a 4% rate of HLH/MAS in this population [42]. The prescribing information for Abecma alluded to a difference in the incidence of HLH depending on the CAR-T cell dose, with HLH/MAS occurring in 8% of patients in the 450 × 10^6^ dose cohort and 1% in the 300 × 10^6^ dose cohort [42]. Initial outcome reports from the phase 3 studies of this product do not currently report adverse events of HLH, MAS or IEC-HS [43]. The prescribing information for ciltacabtagene autoleucel (Carvykti) notes that fatal HLH occurred in 1 patient 99 days after infusion and was preceded by prolonged CRS (Table 3) [44]. Though not presenting specific diagnostic criteria, the prescribing information for both of the approved BCMA-directed CAR-T cell products considered hypotension, hypoxia, multiple organ dysfunction, renal dysfunction and cytopenia as manifestations of HLH/MAS. The Carvykti manufacturers also warned that patients who develop HLH/MAS have an increased risk of severe bleeding and recommend monitoring hematological parameters and transfusing per the institutional guidelines. Further studies of this agent do not yet report adverse events of HLH, MAS or IEC-HS but are notable for the emergence of secondary hematologic malignancies in treated patients [45,46].

At the time of this review, there are various phase 1 and 2 clinical trials for CAR-T products targeting CD22 alone or as a bispecific antibody (e.g., a CD22/CD19 dual-targeting CAR-T cell); none have been FDA-approved yet [47]. However, initial trial reports and retrospective reviews of the outcomes using these products do note the incidence of IEC-HS, which for some CD22-targeted products, can be as high as 40%, which is evidently more frequent than in CD19-targeted products despite a potential decrease in the incidence of CRS and ICANS [34,48,49]. The reasons behind this difference are under investigation; however, increased endothelial activation, immune cell dysregulation (e.g., NK cell lymphopenia) as well as cytokine production (e.g., IL-1, IFN-gamma, IL-18) have been described after the use of CD22 CAR-T products and could play a role in the higher incidence of this hyperinflammatory condition [48].

IEC-HS and other severe toxicities are likely to vary between CAR-T products, dosing strategies and antigen targets. The current criteria include general parameters to describe the clinical manifestations associated with IEC-HS after CAR-T; further research is needed to know whether these criteria should be tailored to the targeted marker, construct or treated condition. Overall, clear guidance regarding the identification, classification and optimal treatment of IEC-HS will be increasingly relevant and helpful to providers as CAR-T products with new targets enter the market and clinical practice.

## 5. Management and Treatment of IEC-HS

### 5.1. Supportive Care

Once IEC-HS has been identified, as with other severe inflammatory CAR-T toxicities (CRS and ICANS), optimal supportive care and monitoring for end organ complications are paramount. Clinical manifestations can include fever, cytopenias, respiratory distress, vasculopathy, coagulopathy, shock and high-grade end organ damage including but not limited to the liver, kidneys, lungs and brain. In the course of these toxicities, patients must have the appropriate level of supportive care, including close monitoring of vital signs, volume status, mental status, and frequent laboratory studies, including a complete blood count, liver and renal function testing as well as coagulation studies, including fibrinogen [16]. Patients can quickly deteriorate with worsening shock refractory to fluids and requiring vasopressors, respiratory distress with escalating oxygen requirements, or neurologic manifestations that require admission to an intensive care unit for monitoring and aggressive management. CT and MRI imaging can help better characterize and identify toxicities such as pulmonary edema or hepatosplenomegaly and assess their response to therapy.

### 5.2. Corticosteroids

Systemic corticosteroids alone or in combination with other anti-inflammatory agents are foundational to various proposed initial and subsequent treatment options for IEC-HS and other inflammatory complications of CAR-T therapy. The use of systemic corticosteroids for the management of CRS and the inflammatory complications of CAR-T has not been demonstrated to undermine treatment efficacy in B-cell lymphomas [50,51]. Early recommendations on the dosing for IEC-HS included its use in combination with cytokine-directed agents, with the dosing correlating with the grade of concomitant CRS or other end organ toxicity. Dexamethasone 10 mg IV every 6 h with escalation to 20 mg IV every 6 h in refractory cases with prompt tapering has been proposed in patients without high-grade end organ damage, concomitant neurotoxicity or refractory grade 4 hypotension [16]. Higher “pulse dosing” has been considered in patients with secondary HLH in the setting of cancer treatment or IEC-HS with refractory grade 4 CRS or end organ toxicities up to methylprednisolone 1–2 mg/kg or dexamethasone 5–10 mg/m^2^ every 6–24 h [16,17,52]. The use of prophylactic corticosteroids was evaluated in cohort 6 of the ZUMA 1 study and showed a decrease in severe toxicities, including grade 3 or higher CRS and ICANS. At 8.9 months of follow-up, the overall response rate was 95% and complete response rate was 80%, which is comparable to previous outcomes in patients who did not receive corticosteroids, suggesting that the prophylactic use of corticosteroids after CAR-T does not decrease response to treatment or negatively impact the CAR-T cell pharmacokinetics [53]. Though not specifically evaluated, it would be reasonable to hypothesize this strategy may also decrease the incidence of IEC-HS. The optimal timing, dosing and tapering strategies may vary significantly depending on treatment response, patient morbidities and risk factors and concomitant therapeutic interventions. The dosing considerations in combination with existing and novel agents will remain an area that needs be addressed in further guidelines as more clinical outcome data become available.

### 5.3. Interleukin-1 (IL-1) Targeting Agent

A growing body of data for the use of Anakinra, an IL-1 receptor antagonist, in various hyper-inflammatory diseases is emerging given its potential to disrupt cytokine storming syndromes with mechanistic similarities to HLH/MAS [54]. While this agent was approved for the treatment of rheumatologic disorders, promising data from off-label use in adults and children have brought it forward into the recommended treatment options in HLH/MAS per the consensus of the Histiocyte Society [55]. Retrospective studies using anakinra in secondary HLH are very encouraging with overall response rates as high as 80–90% [56,57]. In addition, case series have alluded to its efficacy in IEC-HS in patients treated with CD19 CAR-T products for pediatric B-ALL, DLBCL and mantle cell lymphoma [58,59].

Anakinra allows for significant dose escalation and titration with well-established toxicity and tolerability data due to its extensive study in rheumatologic disorders. This treatment can be given IV or subcutaneously (SQ) without significant dosing adjustments in adults. The proposed dosing options include 100–200 mg IV or SQ every 6–12 h in adults or up to 10 mg/kg IV daily, or 4 mg/kg IV every 6 h in pediatric patients. Considering its potential benefits, the ASTCT IEC-HS committee advises consideration of its use early in the course of IEC-HS with or without corticosteroids [17].

### 5.4. JAK1/JAK2 Inhibitors

Ruxolitinib (Jakafi) is a Jak1/Jak2 inhibitor with clinical applications in various hematologic and inflammatory disorders. It has been approved for the treatment of hematological malignancies, including myelofibrosis and polycythemia vera, as well as steroid-refractory graft-vs.-host disease. Various cytokine receptors, including many of the pro-inflammatory cytokines implicated in the development of HLH and IEC-HS, such as IL-2, IL-6, and interferon-gamma (IFN-γ), utilize the JAK/STAT signaling pathways to initiate the gene transcription necessary to propagate further inflammation [60]. Clinical application of this medication capable of modulating a myriad of signaling pathways has recently shown clinical efficacy in refractory HLH [61,62]. Case reports and retrospective studies now demonstrate its clinical efficacy as a first-line treatment for primary and secondary HLH [63,64,65]. According to a 2021 review regarding the use of ruxolitinib in children and adult patients with primary and secondary HLH, there have been at least 115 patients published in the literature that have been treated in this setting, with the majority showing some degree of response [66]. There is well-established safety data in children and adults with a recommended dosing of up to 10–20 mg daily in patients at least 14 years old and a weight-based dosing of 2.5–5 mg daily in children under age 14. The most recent ASTCT guidance recommends the initiation of ruxolitinib in addition to corticosteroids and escalating the dosing of IL-1 blockade with anakinra as a second-line option for IEC-HS [17]. Judicious use in the setting of CAR-T-associated pancytopenia is needed.

### 5.5. Interleukin-6 (IL-6) Inhibition

The monoclonal antibody tocilizumab disrupts downstream IL-6 signaling by binding to the IL-6 receptors, which may interrupt the pro-inflammatory signaling inherent to the development of CRS [67]. Despite strong evidence for its use in CRS, small studies attempting to characterize its effect in IEC-HS trouble the notion of its use in the absence of other active inflammatory complications. The majority of patients who meet the proposed diagnostic criteria for IEC-HS will meet or have met the diagnostic criteria for CRS in their course of treatment. Furthermore, retrospective evaluation of the treatment of secondary HLH has raised concern due to the increased incidence of infectious complications without significant clinical benefit [68]. At the time of this review, there was no substantive data regarding the efficacy of alternative IL-6-pathway-directed agents such as Siltuximab, Sarilumab or Satralizumab in the treatment of IEC-HS.

Interestingly, some studies have even noted an almost paradoxical association between exposure to tocilizumab and the subsequent development of macrophage activation syndrome [68,69]. Other cytokines such as IL-18 have been implicated in the pathogenesis of IEC-HS and have been found to be elevated even after the administration of tocilizumab [48,69,70,71]. While there are no approved agents at this time, IL-18 may be an alternative target of further cytokine-directed therapeutic inventions for IEC-HS.

Another novel approach known as cytokine absorption is used to decrease IL-6 and other cytokine levels using extracorporeal filtration techniques in severe sepsis. Experience with this intervention in HLH is limited to case reports. Its potential role in IEC-HS has yet to be evaluated and is unclear at this time [72,73].

### 5.6. IFN-γ Blockade

Emapalumab is a monoclonal antibody that binds to circulating and receptor-bound IFN-γ, another inflammatory cytokine found to be significantly elevated in primary HLH, IEC-HS and other CAR-T-associated toxicities [34,74]. Emapalumab is now FDA-approved for primary HLH in children and adults in the setting of refractory, recurrent or progressive disease or intolerance to other standard treatments. The initial dosing is a twice-weekly IV infusion of 1 mg/kg. Currently, there are very limited data in the literature pertaining to its efficacy specifically in IEC-HS. Substantial evidence in animal modeling exists for its potential utility in managing CAR-T toxicity without inhibition of the destruction of targeted lymphoma cells [75]. Notable case reports describe the clinical recovery of patients with severe IEC-HS and CRS who received emapalumab in addition to corticosteroids and multiple interleukin-directed treatments [76,77]. This mechanistically promising drug and therapeutic target will likely feature prominently in upcoming studies in refractory IEC-HS. It can be considered for use as a salvage option in patients with high-grade IEC-HS, refractory to anti-cytokine treatments and corticosteroids.

### 5.7. Cytotoxic Anti-T Cell Therapies

Cytotoxic therapies directed toward T cells remain treatment options in cases refractory to corticosteroids and cytokine-directed therapies. The topoisomerase inhibitor etoposide is known to ablate activated T cells and dampen cytokine secretion with evidence of its efficacy in primary HLH in children, as well as primary and secondary HLH in adults [78,79]. The standard dosing in adults would be 150 mg/m^2^ twice weekly with proposed dosing modifications including a reduction in frequency to once weekly and/or dose reduction to 50–100 mg/m^2^ [52]. Etoposide has been used in multi-modal regimens alongside corticosteroids, cyclosporine A and intrathecal treatments in pediatric primary HLH such as the HLH-94 protocol with promising effect [79]. Antithymocyte globulin (ATG) and alemtuzumab have been proposed as salvage therapy options in primary pediatric HLH, but at this time do not have efficacy data regarding their use in IEC-HS [80,81]. The use of these and other anti-T-cell therapies must be considered judiciously in light of the significant clinical instability and end organ damage that can occur in IEC-HS, which may be further exacerbated by the toxicity and immunosuppressive effects of these agents. Patients with severe IEC-HS refractory to initial treatments may derive benefit from cytotoxic anti-T-cell drugs as salvage therapy.

### 5.8. Treatment Considerations and Advancements in IEC-HS

Investigation into the pathophysiology and biochemical pathways of the hyper-inflammatory toxicity generated by CAR-T cells can serve to better inform both initial supportive care and the development of novel therapies for IEC-HS with distinct targets along the implicated inflammatory pathways. Guidance regarding the optimal therapeutic interventions for cellular therapy toxicities including IEC-HS have been developed, refined and made expediently available via CARTOX and recently by an ASTCT expert panel [16,17]. These recommendations leverage therapeutic interventions with known roles in CRS and/or prior HLH disorders. There is acknowledgement that for many patients, these strategies may not have profound clinical effect or mechanistic evidence and that this gap should be addressed by novel therapeutic options [16]. The efficacy, side effect profiles and cumulative immunosuppressant complications of these therapeutic agents may have variations between adult and pediatric populations. All proposed therapeutic interventions have some degree of immune modulatory effect and thus monitoring for bacterial, viral, fungal and other infectious complications is essential.

**Figure 1:**
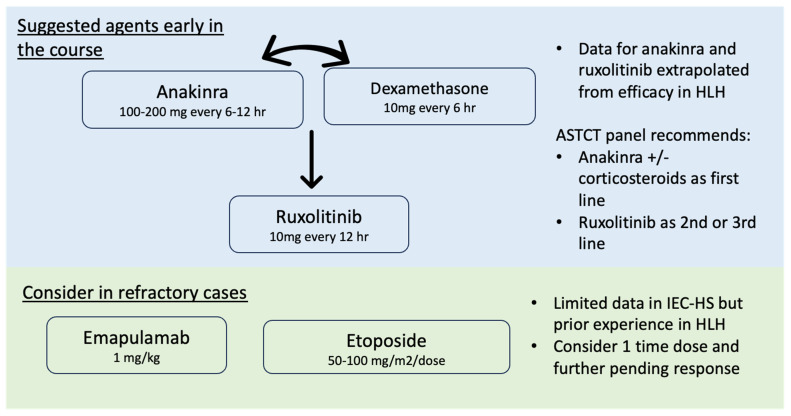
Treatment options for IEC-HS.

The body of evidence for existing treatments in IEC-HS is growing and has been mostly extrapolated from prior experience in primary and secondary HLH (Figure 1). Systemic corticosteroids alone or in combination with other agents remain a mainstay of various proposed treatment strategies. Knowledge regarding the efficacy of cytokine-directed agents including anakinra and ruxolitinib in children and adults is rapidly evolving and appears promising, favoring its use early in the course of IEC-HS. Further lines of treatment with emapalumab and/or cytotoxic anti-T-cell agents may be considered in refractory cases, although data in IEC-HS are lacking. The utilization of other novel anti-inflammatory agents and treatment strategies for HLH that may be applicable in IEC-HS is an area of ongoing study [67].

## 6. Conclusions

As the role of CAR-T cell therapy expands, patients and providers will more frequently face the challenges posed by its unique toxicities, such as IEC-HS. Initially labeled as secondary HLH and MAS, these terms have now been supplanted by this more mechanistic description. Reconciliation of the latest terminology and classification system with those previously utilized in initial clinical trials and studies of the toxicities of CAR-T cell therapy is important in assessing the true prevalence and severity of IEC-HS in the existing literature. Research has only recently turned to addressing the genetic features and biochemical signaling pathways most relevant to this hyper-inflammatory toxicity. Accordingly, guidelines on initial identification, diagnostic criteria, treatment and the management of refractory cases continue to evolve. Currently, the treatment options supported by the data extrapolated from HLH include corticosteroids, cytokine-directed therapy and cytotoxic T cell agents; emergence of further clinical trial results and post-marketing data can be leveraged to expand our understanding and inform further strategies for prevention and treatment. Continued efforts toward unifying the prior and current terminology, diagnostic criteria and treatment guidelines will be essential to providers for the optimization of patient care and outcomes for this often fatal complication of CAR-T cell therapy.

## Figures and Tables

**Table 1:** Evolution of diagnostic criteria for secondary HLH/IEC-HS.

HLH-94 criteria (1994) [13]	- Fever - Splenomegaly - Bicytopenia - Hypertriglyceridemia and/or hypofibrinogenemia - Hemophagocytosis
HLH-2004 criteria (2004) [14]	Must have 5 of the following: - Fever ≥ 38.5 °C - Splenomegaly - Bicytopenia - Hemoglobin < 9 g/dL - Platelets < 100,000/uL - Absolute neutrophil count < 1000/uL - Hypertriglyceridemia > 265 mg/dL and/or hypofibrinogenemia < 150 mg/dL - Hemophagocytosis in bone marrow, spleen, lymph node or liver - Hyperferritinemia > 500 ng/mL - Low/absent NK-cell-activity - Elevated soluble interleukin-2-receptor levels
H score [15] (2014)	Points assigned as following: - Immunosuppression (+18 points) - Temperature - <101.1 °F (<38.4 °C) (0 points) - 101.1–102.9 °F (38.4–39.4 °C) (+33 points) - >102.9 °F (>39.4 °C) (+49 points) - Organomegaly - No (0 points) - Hepatomegaly or splenomegaly (+23 points) - Hepatomegaly and splenomegaly (+38 points) - Number of cytopenias - 1 lineage (0 points) - 2 lineages (+24 points) - 3 lineages (+34 points) - Ferritin - <2000 ng/mL (0 points) - 2000–6000 ng/mL (+35 points) - >6000 ng/mL (+50 points) - Triglycerides - <132.7 mg/dL (0 points) - 132.7–354 mg/dL (+44 points) - >354 mg/dL (+64 points) - Fibrinogen - >250 mg/dL (0 points) - <250 mg/dL (+30 points) - AST - <30 (0 points) - >30 (+19 points) - Hemophagocytosis on bone marrow aspirate - No (0 points) - Yes (+35 points) Score correlates with percent likelihood of HLH with higher points indicating higher likelihood of HLH. Best cut-off value was 169 points
CARTOX criteria [16] (2017)	- Ferritin levels peak at >10,000 ng/mL during the CRS phase - Any two of the following: - Grade 3 or greater liver toxicity - Grade 3 or greater kidney toxicity - Grade 3 or greater lung toxicity - Hemophagocytosis in the bone marrow or other organs
ASTCT criteria [17] (2023)	Most common manifestations: - Required: elevated ferritin (>2 × ULN or baseline and/or rapidly rising) - Onset with resolving/resolved CRS or worsening inflammatory response after initial improvement with CRS-directed therapy - Hepatic transaminase elevation (>5 × ULN or >5 × baseline) - Hypofibrinogenemia (<150 mg/dL or <LLN) - Hemophagocytosis in bone marrow or other tissue - Cytopenias Other manifestations that may be present - Lactate dehydrogenase elevations (>ULN) - Other coagulation abnormalities (e.g., elevated PT/PTT) - Direct hyperbilirubinemia - New-onset splenomegaly - Fever (new or persistent) - Neurotoxicity - Pulmonary manifestations (e.g., hypoxia, pulmonary infiltrates, pulmonary edema) - Renal insufficiency (new onset) - Hypertriglyceridemia (fasting level, >265 mg/dL)

**Table 2:** Prescribing information from manufacturers for CD-19 CAR-T products.

Product	Trial	Indication	Incidence of HLH/MAS
Tisagenlecleucel (brand name: Kymriah)	ELIANA [18]	B-ALL (peds/AYA)	6% (5/79)
	JULIET [19]	Large B-cell lymphoma	2% (2/115)
	ELARA [20]	Follicular lymphoma	1% * (1/97)
Axicabtagene ciloleucel (brand name: Yescarta)	ZUMA-7 [21]	Large B-cell lymphoma	0% (0/168)
	ZUMA-1 [22]	Large B-cell lymphoma	1% (1/108)
	ZUMA-5 [23]	Indolent non-Hodgkin lymphoma (124 follicular lymphoma, 22 marginal zone)	0% (0/146)
Brexucabtagene autoleucel (brand name: Tecartus)	ZUMA-2 [24]	Mantle cell lymphoma	0% (0/82)
	ZUMA-3 [25]	B-ALL	4% (3/78)
lisocabtagene maraleucel (brand name: Breyanzi)	Transform [26]	Large B-cell lymphoma	1.1% (1/89
	PILOT [27]	Large B-cell lymphoma	0% (0/61)
	TRANSCEND [28]	Large B-cell lymphoma	0% (0/268)

* One patient (1%) with r/r follicular lymphoma developed HLH > 1 year after receiving Kymriah with a fatal outcome. The patient did not have CRS during or immediately preceding HLH.

**Table 3:** Prescribing information from manufacturer for other CAR-T products.

Product	Target Antigen	Trial	Indication	Incidence of HLH/MAS
Idecabtagene vicleucel (brand name: Abecma)	BCMA	KarMMa [42]	Multiple myeloma	4% (5/127)
Ciltacabtagene autoleucel (brand name: Carvykti)	BCMA	CARTITUDE-1 [44]	Multiple myeloma	1% (1/97) *

* Also mentioned 1 patient with grade 4 HLH/MAS developed fatal intracerebral and gastrointestinal hemorrhage in the setting of coagulopathy and thrombocytopenia 12 days after treatment in another ongoing study.
